# Vasoactive Biomarkers Associated With Long-Term Incidence of Symptomatic Peripheral Arterial Disease and Mortality

**DOI:** 10.1177/0003319720987739

**Published:** 2021-01-28

**Authors:** Ardwan Dakhel, Gunnar Engström, Olle Melander, Stefan Acosta, Shahab Fatemi, Anders Gottsäter, Moncef Zarrouk

**Affiliations:** 1 Department of Clinical Sciences, Malmö, 5193 Lund University, Sweden; 2 Department of Cardiothoracic and Vascular Surgery, Malmö, Sweden; 3 Department of Internal Medicine and Emergency Medicine, Malmö, Sweden

**Keywords:** peripheral arterial disease, biomarkers, atherosclerosis

## Abstract

We evaluated if plasma biomarkers can predict incident peripheral arterial disease (PAD) and mortality in a longitudinal cohort study. Men (n = 3618) and women (n = 1542) were included in the Malmö Preventive Project and underwent analysis of: C-terminal endothelin-1 (CT-proET-1), N-Terminal prosomatostatin (NT-proSST), midregional proatrial natriuretic peptide (MR-proANP), procalcitonin (PCT), and copeptin. Participants were followed up for incident PAD and mortality until December 31, 2016. Median follow-up was 11.2 years (interquartile range 9.4-12.2). Cumulative incidence of PAD was 4.3% (221/5160), 4.5% in men (164/3618) and 3.7% in women (57/1542; *P* = .174). In an adjusted Cox proportional hazards regression model, higher CT-proET-1 (hazard ratio [HR] 1.8; 95% confidence interval [CI] 1.4-2.3), NT-proSST (HR 1.5; 95% CI 1.2-2.0), and MR-proANP (HR 1.7; 95% CI 1.3-2.3) were independently associated with incident PAD, and higher CT-proET-1 (HR 1.3; 95% CI 1.2-1.5), NT-proSST (HR 1.2; 95% CI 1.1-1.3), MR-proANP (HR 1.4; 95% CI 1.3-1.6), PCT (HR 1.1; 95% CI 1.0-1.2), and copeptin (HR 1.2; 95% CI 1.1-1.4) were independently associated with mortality. Increased levels of CT-proET-1, NT-proSST, and MR-proANP were independently associated with incident PAD, whereas all the vasoactive biomarkers were independently associated with mortality during follow-up.

## Introduction

Peripheral arterial disease (PAD) of the lower extremities presents as intermittent claudication or critical limb ischemia decreasing quality of life.^1,2^ Peripheral arterial disease was reported to affect 202 million people worldwide in 2010, and its prevalence increases with age.^2^ Peripheral arterial disease is associated with a high risk of all-cause mortality and cardiovascular (CV) morbidity, emphasizing the need for prompt initiation of preventive measures (eg, smoking cessation and lipid-lowering and antithrombotic agents).^2^ Current screening methods are based on measuring the ankle-brachial index (ABI) and clinical evaluation. Limitations of ABI are inaccurate measurements in patients with severely calcified arteries often present in old age and in patients with diabetes or renal insufficiency.^3^

The complex pathophysiology of PAD involves an inflammatory, atherosclerotic, and hypercoagulable state contributed to by vascular endothelial cells, vascular smooth muscle cells, and inflammatory cells.^4^ This process caused by endothelial and macrovascular dysfunction suggests a role for CV biomarkers.

C-terminal endothelin-1 (CT-proET-1) is a precursor of endothelin-1 (ET-1), a peptide with vasoconstrictive properties playing a role in the pathogenesis of atherosclerosis and peripheral endothelial dysfunction.^5^

N-Terminal prosomatostatin (NT-proSST) is a fragment of the somatostatin precursor. Somatostatin inhibits the release of growth hormone and insulin-like growth factor-1, and the secretion of glucagon. Recent studies have shown that elevated levels of NT-proSST are associated with an increased incidence of CV disease (CVD) and all-cause mortality.^6^

Midregional proatrial natriuretic peptide (MR-proANP) is a precursor of atrial natriuretic peptide, which is potentially associated with microvascular endothelial dysfunction.^7^ Elevated MR-proANP levels have been cross-sectionally related to PAD.^8^

Procalcitonin (PCT) participates in monocyte adhesion and migration, which has stimulatory effects on nitric oxide synthase gene expression in sepsis.^9^

Copeptin is the C-terminal fragment of pro-arginine vasopressin (AVP), a peptide released in response to several inflammatory stimuli. Copeptin acts as a nonspecific marker of acute illness, and disease severity with studies showing elevated plasma concentrations in acute CV conditions, such as myocardial infarction (MI).^10^ Major risk factors for PAD resemble those for coronary and cerebrovascular disease.^11^ The aim of the present study was to evaluate if biomarkers associated with other CVD can predict incident PAD and mortality during long-term follow-up.

## Material and Methods

### Study Population

**Figure 1.**
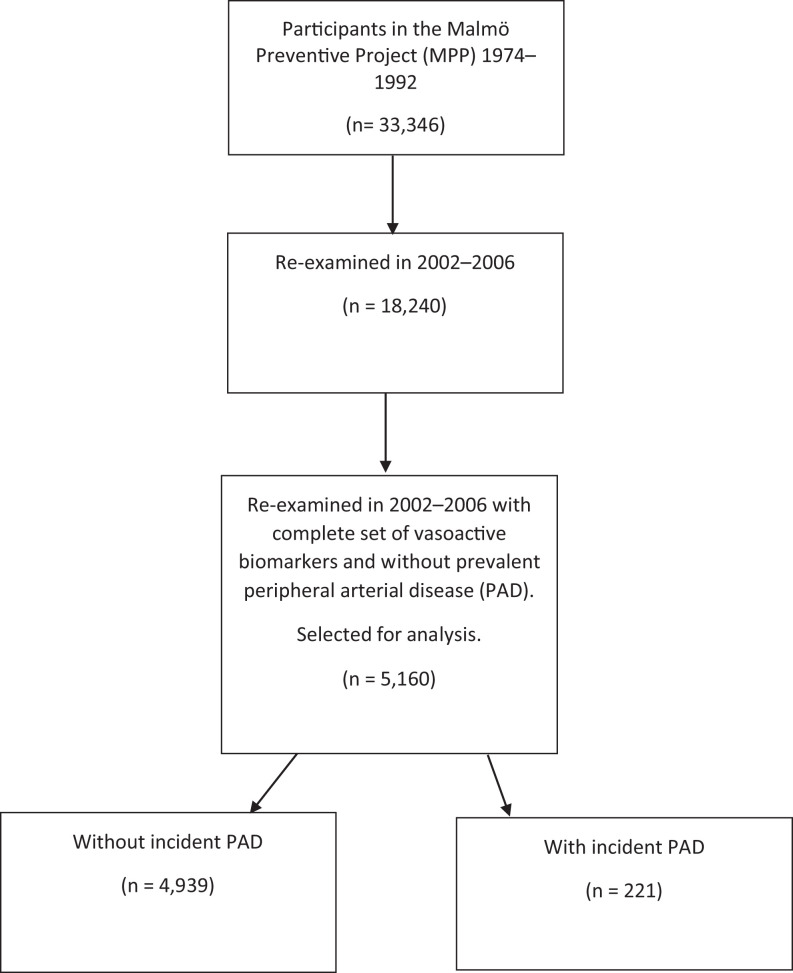
Selection of study participants.

In the population-based Malmö Preventive Project (MPP), 22 444 men and 10 902 women, with a homogenous ethnic background from Malmö, Sweden, were recruited between 1974 and 1992 to participate in a health screening program. During 2002 to 2006, all surviving participants were invited for reexamination; 18 240 participants participated. Here, CV disorders and risk factors were reassessed, and plasma was frozen to −80°C for later analyses. Complete analysis of vasoactive biomarkers was performed in 5160 participants without prevalent PAD at the time of reexamination (Figure 1). In this group, biomarkers were related to incident PAD and mortality. Detailed information on methodological aspects and the main results from MPP have been published.^12^

### Clinical Examination and Assays

Participants underwent medical history, physical examination, self-administered questionnaire, and laboratory assessment. All participants were examined for height (m) and weight (kg), and body mass index (BMI) was calculated in kg/m^2^. Systolic blood pressure (SBP) and diastolic blood pressure (DBP) (mm Hg) were measured twice in the supine position after 10 minutes rest and a mean figure was recorded. Serum (s-) total cholesterol (mmol/L), s-triglycerides (mmol/L), and data from an oral glucose tolerance test (mmol/L) were analyzed using routine methods at the Department of Clinical Chemistry, Skåne University Hospital. A self-administered questionnaire was used to evaluate smoking. “Ever smoker” was defined as a participant who has been smoking on a daily basis for ≥6 months.^12^ Diabetes mellitus was defined as fasting plasma glucose ≥7.0 mmol/L.

Plasma biomarkers were measured in fasting blood samples that had been frozen at −80°C. Circulating levels of CT-proET-1, MR-proANP, and copeptin were assessed using the following assays according to the manufacturer’s instructions: Thermo Scientific B·R·A·H·M·S CT-proET-1 KRYPTOR, Thermo Scientific B·R·A·H·M·S MR-proANP KRYPTOR, and Thermo Scientific B·R·A·H·M·S CT-proAVP LIA (B·R·A·H·M·S).^7^ To estimate the plasma level of NT-proSST, the chemiluminescence/coated tube format (B·R·A·H·M·S GmbH) was used with a detection limit of 4 pmol/L. The interlaboratory coefficient of variation was 20% at 18 pmol/L, 10% at 50 pmol/L, and <6% for above 100 pmol/L. The native analyte stability was tested in EDTA plasma from 10 different individuals at 22 °C and 37 °C.^13^

### Follow-Up and End Point Retrieval

The 5160 participants with a complete set of vasoactive biomarkers at the time of reexamination were followed for all-cause mortality and incident PAD until December 31, 2016 (Figure 1). Participants with a first registered diagnosis of PAD were identified from Swedish national registers (the Inpatient and Outpatient Register and the Cause of Death Register) by linkage of the 10-digit personal identification number unique to each Swedish resident.^14^ In both validated registers, diagnoses are coded using a Swedish revision of the *International Statistical Classification of Diseases and Related Health Problems* (*ICD*), version 8 (443,90; 443,99; 440,20; 445,00; 445,98; 445,99), 9 (443X; 440C), 10 (I73.9 [all sub codes except I73.9A]; I70.2 [all sub codes]).^14^ Codes for embolism to the lower extremity (444,20, 444; I74.3, respectively) were excluded. Previous validation of PAD diagnoses in Swedish national registries in records at our hospital has documented a validity of 98%.^15^

The study was approved by the previous regional research committee in Lund, Sweden, 2014/643. The investigation conforms to the principles outlined in the Declaration of Helsinki.

### Statistics

Quantitative normal and skewed distributed variables are presented as mean with standard deviation (SD) and median with interquartile range (IQR), respectively. Dichotomous variables are presented as count and proportion. Differences between groups were assessed with the Mann-Whitney *U* test for continuous nonparametric variables, *t* test for continuous parametric variables, and χ^2^ test for nominal variables. The end point studied in subsequent prospective analyses was incident PAD and mortality. Due to skewed distributions, log transformed values of CT-proET-1, NT-proSST, MR-proANP, PCT, and copeptin were used in the Cox proportional hazards regression models. Hazard ratios (HRs) with 95% confidence intervals (CI) were expressed per 1 SD increment of each respective log transformed plasma biomarker in the Cox proportional hazard model. Risk factors for PAD (age, gender, BMI, diabetes mellitus, ever smoking, cholesterol, low density lipoprotein, high density lipoprotein, SBP, DBP) and plasma biomarkers were included in a multivariable-adjusted model. Each biomarker was entered separately into the model together with all risk factors. The survival analysis was analyzed with the Kaplan-Meier estimator. A 2-sided *P* < .05 was considered significant. Analyses were performed using SPSS for Windows, version 25.0 (SPSS Inc).

## Results

### Background Characteristics

Median follow-up was 11.2 years (IQR 9.4-12.2). The overall cumulative incidence of PAD was 4.3% (221/5160), 4.5% in men (164/3618) and 3.7% in women (57/1542) (*P* = .174). Baseline risk factor characteristics for participants with or without incident PAD are shown in Table 1.

**Table 1.** Baseline Characteristics in Participants Without and With Incident PAD During Follow-Up.^a^

Characteristic	No PAD (n = 4939)	Incident PAD (n = 221)
Age (years)	68.3 (66.5-74.5)	68.50 (67.2-74.7)
Gender, n (% men)	3454 (69.9%)	164 (74.2%)
BMI (kg/m^2^)	25.9 (23.7-28.7)	26.2 (23.1-28.4)
Diabetes mellitus, n (%)	543 (11.0%)	50 (22.6%)
Ever smoking, n (%)	2386 (48.3%)	148 (67.0%)
Total cholesterol (mmol/L)	5.5 (4.8-6.3)	5.5 (4.7-6.1)
Triglycerides (mmol/L)	1.1 (0.8-1.5)	1.2 (0.9-1.7)
LDL cholesterol (mmol/L)	3.6 (2.9-4.3)	3.5 (2.6-4.2)
HDL cholesterol (mmol/L)	1.3 (1.1-1.6)	1.2 (1.0-1.5)
Glucose (mmol/L)	5.5 (5.1-6.1)	5.7 (5.2-6.6)
Systolic blood pressure (mm Hg)	144.0 (131.0-158.0)	149.0 (135.5-162.5)
Diastolic blood pressure (mm Hg)	84.0 (76.0-91.0)	83.0 (77.0-90.0)
CT-proET-1 (pmol/l)	67.5 (59.5-77.7)	72.9 (63.0-85.3)
NT-proSST (pmol/l)	436.5 (350.0-559.0)	502.0 (404.5-665.5)
MR-proANP (pmol/l)	103.1 (76.0-144.0)	119.4 (86.0-180.3)
PCT (µg/l)	0.035 (0.026-0.047)	0.038 (0.028-0.052)
Copeptin (pmol/l)	7.2 (4.4-12.0)	7.4 (4.5-13.5)

Abbreviations: BMI; body mass index; CI; confidence interval; CT-proET-1, C-terminal endothelin-1; HDL, high-density lipoprotein; LDL, low-density lipoprotein; MR-proANP, midregional proatrial natriuretic peptide; NT-proSST, N-Terminal prosomatostatin; PAD, peripheral arterial disease; PCT, Procalcitonin.

^a^ Median and interquartile range (if not otherwise specified).

### Biomarkers and Incident PAD

In the adjusted Cox proportional hazards regression model, higher CT-proET-1 (HR 1.8; 95% confidence interval [CI] 1.4-2.3), NT-proSST (HR 1.5; 95% CI 1.2-2.0), and MR-proANP (HR 1.7; 95% CI 1.3-2.3) were independently associated with incident PAD (Table 2).

**Table 2.** Cox Regression Analysis of Plasma Biomarkers at Baseline and the Risk of Incident PAD During Follow-Up.^a^

Variables	β	HR^b^ (95% CI) for incident PAD	*P*
CT-proET-1	0.6	1.8 (1.4-2.3)	**<.001**
NT-proSST	0.4	1.5 (1.2-2.0)	**.002**
MR-proANP	0.5	1.7 (1.3-2.3)	**.001**
PCT	0.1	1.1 (0.9-1.4)	.380
Copeptin	0.2	1.2 (0.9-1.7)	.207

Abbreviations: BMI; body mass index; CI; confidence interval; CT-proET-1, C-terminal endothelin-1; HDL, high-density lipoprotein; LDL, low-density lipoprotein; MR-proANP, midregional proatrial natriuretic peptide; NT-proSST, N-Terminal prosomatostatin; PAD, peripheral arterial disease; PCT, Procalcitonin.

^a^ The following biomarkers were entered in the multivariable analysis besides each respective plasma biomarker and adjusted for: age, gender, BMI, diabetes mellitus, ever smoking, cholesterol, LDL, HDL, triglycerides, systolic blood pressure, diastolic blood pressure.

^b^ Hazard ratio (HR) expressed per 1 standard deviation (SD) increment of each respective log transformed plasma biomarker in the Cox proportional hazard model.

### Biomarkers and Mortality

Median survival was 11.2 years (IQR 9.6-12.2) in participants without incident PAD, and 10.9 years (IQR 8.0-12.1) in participants with incident PAD (*P* < .001). During follow-up, 1371/3618 (40%) men died compared to 421/1542 (27%) women (*P* < .001).

In the adjusted Cox proportional hazards regression model, higher CT-proET-1 (HR 1.3; 95% CI 1.2-1.5), NT-proSST (HR 1.2; 95% CI 1.1-1.3), MR-proANP (HR 1.4; 95% CI 1.3-1.6), PCT (HR 1.1; 95% CI 1.0-1.2), and copeptin (HR 1.2; 95% CI 1.1-1.4) were independently associated with mortality (Table 3).

**Table 3.** Cox Regression Analysis of Plasma Biomarkers at Baseline and the Risk of Mortality During Follow-Up.^a^

Variables	β	HR^b^ (95% CI) for incident PAD	*P*
CT-proET-1	0.3	1.3 (1.2-1.5)	**<.001**
NT-proSST	0.2	1.2 (1.1-1.3)	**<.001**
MR-proANP	0.3	1.4 (1.3-1.6)	**<.001**
PCT	0.1	1.1 (1.0-1.2)	**.011**
Copeptin	0.2	1.2 (1.1-1.4)	**<.001**

Abbreviations: BMI, body mass index; CI, confidence interval; CT-proET-1, C-terminal endothelin-1; HDL, high-density lipoprotein; LDL, low-density lipoprotein; MR-proANP, midregional proatrial natriuretic peptide; NT-proSST, N-Terminal prosomatostatin; PCT, Procalcitonin; PAD, peripheral arterial disease.

^a^ The following biomarkers were entered in the multivariable analysis besides each respective plasma biomarker and adjusted for: age, gender, BMI, diabetes mellitus, ever smoking, cholesterol, LDL, HDL, systolic blood pressure, diastolic blood pressure.

^b^ Hazard ratio (HR) expressed per 1 standard deviation (SD) increment of each respective log transformed plasma biomarker in the Cox proportional hazard model.

## Discussion

**Figure 2.**
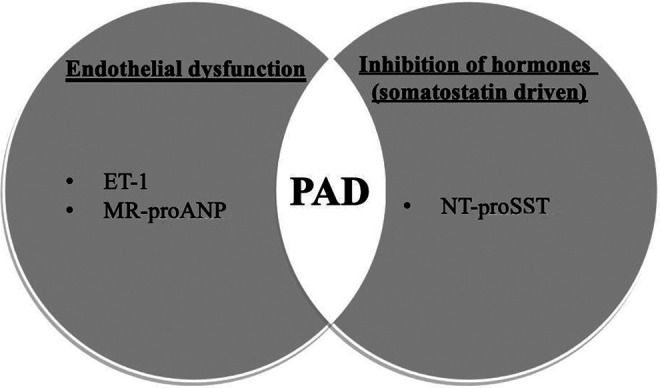
C-terminal endothelin-1 (ET-1), N-Terminal prosomatostatin (NT-proSST), and midregional proatrial natriuretic peptide (MR-proANP), and putative underlying mechanisms in development of PAD.

The present study suggests that CT-proET-1, NT-proSST, and MR-proANP after adjustment for relevant risk factors can identify participants at risk for incident symptomatic PAD (Figure 2). Furthermore, increased levels of CT-proET-1, NT-proSST, MR-proANP, PCT, and copeptin might predict reduced long-term survival.

Our results corroborate data from the Cardiovascular Disease in Intermittent Claudication case–control study, in which associations of different biomarkers and PAD were evaluated. The study showed higher risk of prevalent PAD in participants with elevated CT-proET-1 and MR-proANP concentrations.^8^

To our knowledge, this study is the first to longitudinally evaluate the associations of CT-proET-1, NT-proSST, and PCT with incident PAD and mortality. Associations between MR-proANP, copeptin, and incident PAD during follow-up, on the other hand, have been documented in another study population from Malmö.^15^

Endothelin-1 has been implicated in the development of hypertension, chronic heart failure, and acute MI. By decreasing collagen degradation and contributing to endothelial dysfunction, ET-1 has been shown to decrease large vessel elasticity and increase pulse pressure in hypertensive patients.^5,16^ This effect on the arteries decreases the arterial compliance and could lead to the increase of incident PAD and CVD related mortality. ET-1 has both inhibitory and stimulatory effects on platelets in vivo, but has been shown to attenuate acetylcholine induced inhibition of platelet activity in humans in vivo.^17^

Elevated levels of NT-proSST in participants later developing PAD could be due to the inhibition of key hormones of metabolism induced by somatostatin, influencing the development of hypertension, and hyperglycemia which are major risk factors for PAD and CVD.^18^

Atrial natriuretic peptide has been associated with lower blood pressure due to decreased activity of the renin angiotensin aldosterone system, and has been shown to inhibit endothelial hyperpermeability secondary to different inflammatory stimuli, suggesting a vasoprotective ability.^19^ The increased levels of MR-proANP shown in patients with reduced long-term survival may reflect a physiological response to the inflammatory, and hemodynamic changes typically associated with CVD.

Vasopressin is a peptide released into circulation from the hypothalamic gland as a response to changes in plasma osmolarity, traumatic, and endogenous stress.^20^ The increased levels of copeptin in participants with reduced long-term survival could be a result of atherosclerotic changes, resulting in tissue hypoperfusion, consequent osmotic alteration, and endogenous stress increasing the risk of fatal CV events.^21^

According to current European PAD guidelines,^22^ all patients with PAD are recommended lipid-lowering drugs, smoking cessation, blood pressure control, healthy diet, and exercise. Furthermore, symptomatic PAD patients should be treated with antithrombotic drugs.^22^ As elevated levels of CT-proET-1, NT-proSST, and MR-proANP can help us predict individuals at risk for future PAD development, they might be used to motivate individuals at risk to comply with abovementioned guideline recommendations to counteract risk factors at an early stage. Perhaps best medical treatment with lipid-lowering drugs, blood pressure control, and antithrombotic drugs will result in decreased levels of CT-proET-1, NT-proSST, and MR-proANP, and therefore be used as a prognostic tool regarding incidence PAD.

Mortality during follow-up was increased in participants with incident PAD; however, since we have no data on causes of death, we can only presume that this is related to associated endothelial dysfunction and microvascular changes increasing the risk of fatal CV events. Tasevska et al previously showed that participants from the MPP with increased levels of copeptin had higher incidence of coronary artery disease and higher mortality compared with those with lower values.^23^

The design of the present study has some limitations. We performed a retrospective analysis of a longitudinal cohort study consisting of a rather homogenous population with regard to ethnicity; 83% of the participants were born in Sweden,^12^ which may affect the generalizability of the results. The end point of the study, incident PAD, was not a preset end point. Consequently, ABI and biomarkers were not analyzed at baseline, and identification of incident disease both at baseline and during follow-up was based on ICD codes. Ankle-brachial index measurement at study entry would have been desirable to identify not only symptomatic but also asymptomatic PAD at baseline. Furthermore, we do not have reliable ascertainment of PAD in participants who died or emigrated during follow-up. Throughout the years the classification of PAD has been recorded by different ICD systems, and participants might have made major lifestyle changes which could cause confounding when analyzing the data. Since the start of the MPP, smoking rates in Sweden have declined,^24^ and increased use of lipid-lowering drugs and antithrombotic drugs in the modern era might also have impacted the incidence of PAD and related outcomes.^25^ Additionally, data on medication and renal function at rescreening were not available. Neither did we have data concerning HbA_1c_ or the distribution of diabetes type I and type II, however only 9.4% of patients aged 40 to 75 years with diabetes in Malmö around the study period had type I diabetes.^26^

Since CT-proET-1, NT-proSST, and MR-proANP could further predict individuals at risk for future PAD development, they might be used at an early stage to motivate individuals at risk to comply with a healthier lifestyle and guideline recommendations^22^ to counteract CV-related risk factors. More research work is needed, however, as the analyses are not yet widely available for routine clinical use at reasonable costs and speed.

## Conclusion

Increased levels of CT-proET-1, NT-proSST, and MR-proANP were independently associated with incident PAD, whereas all vasoactive biomarkers were independently associated with mortality during follow-up.
